# Effect of perinatal depression on risk of adverse infant health outcomes in mother-infant dyads in Gondar town: a causal analysis

**DOI:** 10.1186/s12884-021-03733-5

**Published:** 2021-03-26

**Authors:** Abel Fekadu Dadi, Emma R. Miller, Richard J. Woodman, Telake Azale, Lillian Mwanri

**Affiliations:** 1 Department of Epidemiology and Biostatistics, Institute of Public Health, College of Medicine and Health Sciences, University of Gondar, Gondar, Ethiopia; 2 Flinders University, College of Medicine and Public health, Health Sciences Building, Sturt Road, Bedford Park, Adelaide, SA 5054 Australia; 3 Department of Health promotion and Behavioral sciences, Institute of Public Health, College of Medicine and Health Sciences, University of Gondar, Gondar, Ethiopia

**Keywords:** Causal effects, Targeted maximum likelihood estimation, Diarrhea, ARI, Malnutrition

## Abstract

**Background:**

Approximately one-third of pregnant and postnatal women in Ethiopia experience depression posing a substantial health burden for these women and their families. Although associations between postnatal depression and worse infant health have been observed, there have been no studies to date assessing the causal effects of perinatal depression on infant health in Ethiopia. We applied longitudinal data and recently developed causal inference methods that reduce the risk of bias to estimate associations between perinatal depression and infant diarrhea, Acute Respiratory Infection (ARI), and malnutrition in Gondar Town, Ethiopia.

**Methods:**

A cohort of 866 mother-infant dyads were followed from infant birth for 6 months and the cumulative incidence of ARI, diarrhea, and malnutrition were assessed. The Edinburgh Postnatal Depression Scale (EPDS) was used to assess the presence of maternal depression, the Integrated Management of Newborn and Childhood Illnesses (IMNCI) guidelines were used to identify infant ARI and diarrhea, and the mid upper arm circumference (MUAC) was used to identify infant malnutrition. The risk difference (RD) due to maternal depression for each outcome was estimated using targeted maximum likelihood estimation (TMLE), a doubly robust causal inference method used to reduce bias in observational studies.

**Results:**

The cumulative incidence of diarrhea, ARI and malnutrition during 6-month follow-up was 17.0% (95%CI: 14.5, 19.6), 21.6% (95%CI: 18.89, 24.49), and 14.4% (95%CI: 12.2, 16.9), respectively. There was no association between antenatal depression and ARI (RD = − 1.3%; 95%CI: − 21.0, 18.5), diarrhea (RD = 0.8%; 95%CI: − 9.2, 10.9), or malnutrition (RD = -7.3%; 95%CI: − 22.0, 21.8). Similarly, postnatal depression was not associated with diarrhea (RD = -2.4%; 95%CI: − 9.6, 4.9), ARI (RD = − 3.2%; 95%CI: − 12.4, 5.9), or malnutrition (RD = 0.9%; 95%CI: − 7.6, 9.5).

**Conclusion:**

There was no evidence for an association between perinatal depression and the risk of infant diarrhea, ARI, and malnutrition amongst women in Gondar Town. Previous reports suggesting increased risks resulting from maternal depression may be due to unobserved confounding.

**Supplementary Information:**

The online version contains supplementary material available at 10.1186/s12884-021-03733-5.

## Background

Women are particularly vulnerable to depression during pregnancy and childbirth which may partly be attributable to hormonal change, social factors such as luck of support, and economic deprivation for women in low income countries [1–4]. Perinatal depression refers to depression occurring during pregnancy (antenatal depression) and after child birth (postnatal depression) [5]. In Ethiopia, the prevalence of antenatal and postnatal depression has been reported in the range from 7 to 31.1% and 9 to 33.8%, respectively [6–9].

Adequate provision of care for a new-born baby requires commitment and engagement [10]. These qualities can be reduced in depression due to associated fatigue, guilt, loss of concentration, worthlessness or hopelessness [11]. Antenatal depression is associated with a 30% risk of reduced maternal infant responsiveness [12, 13] whilst postnatal depression has been associated with impaired growth and poor cognitive development amongst infants, particularly those in low-income countries [14–16]. Infants of antenatally depressed women in low- and middle-income countries have been found to have an increased risk for early cessation of breastfeeding, stunting and underweight [17–20], although these findings are equivocal [14, 15, 20, 21].

In Ethiopia, the association between maternal common mental disorders and the risk of infant illnesses and malnutrition has been inconsistently observed [22–24]. Differences in findings might potentially be due to bias as a result of unobserved confounding, which cannot be overcome using standard regression techniques [25, 26]. Failure to account for unobserved confounding can weaken the quality of evidence derived from such studies [27].

The current study therefore applied recently developed causal inference techniques to estimate the causal average treatment effect of perinatal depression on the risk of diarrhea, acute respiratory infection (ARI) and malnutrition in infants aged up to 6 months. We used the doubly robust semi-parametric method known as Targeted Maximum Likelihood Estimation (TMLE) [28] which reduces the risk of bias by modelling the exposure as well as the outcome. Such models are robust to bias provided that either the exposure or the outcome model is correctly specified i.e. includes all relevant confounders. We also examined likely mediators of any causal associations.

## Methods

We conducted a community-based cohort study in Gondar Town, Ethiopia. Pregnant women were recruited in their second to third trimester and followed for 6 months (from June 2018 to March 2019) after birth when their infants were assessed for the development of diarrhea, ARI, and malnutrition. Gondar Town is an administrative zone of Amhara Regional State, located 747 km north of Addis Ababa (the capital city of Ethiopia). The town has 12 ‘kebeles’ (the smallest administrative units in the country), and in 2017/2018 had 6450 pregnancies [29, 30]. Gondar town has one government-operated referral hospital, eight health centers and 15 private medical clinics [31].

### Sample size

This analysis forms part of a large mother-child health cohort study designed to examine the incidence and prevalence of perinatal depression and its effects on birth and infant health outcomes. The required sample size was determined using Epi Info version 7 [32], with the following assumptions: a type-1 error rate of alpha = 0.05, 90% power, an exposed to unexposed (perinatal depression) ratio of 1:2, and an odds ratio of low birth weight of 1.5 for infants born to women with depression compared to those without depression. A sample size of 809 was estimated and 20% was added for expected losses during follow up, giving a final recruitment target of 970.

### Ethical approval

Ethics approval was obtained from the Institutional Review Board of the University of Gondar and the Social and Behavioral Research Ethics Committee (SBREC) of Flinders University in South Australia [33]. A support letter was provided by the mayor’s office for Gondar town. Participants were informed of the study’s aims and objectives and their right to withdraw from the research during follow-up. Each volunteer was asked to provide written consent and confidentiality was maintained throughout the study. Women with an overall Edinburgh Postnatal Depression Scale (EPDS) score of 13 to 16, and a score 1, 2, or 3 on item ten (thought of suicide) were referred to University of Gondar Specialized Hospital [34] for further diagnosis and treatment, whilst those with an overall EPDS ≥17 were excluded from the study.

### Data collection and the questionnaire

Trained nurse data collectors conducted face-to-face interviews with women in their home using a structured electronic-based questionnaire (supplementary material 1) to collect data on the exposure, outcomes, and potential confounders. The Open Data Collection Kit (ODK) was used to collect the data online using a Lenovo 7 tablet after being checked for validity using Enketo [35] and uploaded to the Google cloud platform.

### Exposures

The Edinburgh Postnatal Depression Scale (EPDS) developed by Cox [34] and adapted for use in the Ethiopian context [36] was used to assess the mother’s depression. The tool measures the extent of stress that pregnant women experienced during the previous week [37–39] and has been validated in the urban population with a sensitivity, specificity, and misclassification rate of 78.9, 75.3, and 24.0% respectively. Women were considered to be depressed if they had an EPDS score of ≥12 during pregnancy (antenatal depression) and ≥ 6 during the postnatal period (postnatal depression) [40]. The Cronbach’s alpha for internal consistency was 0.74 in this study.

### Outcomes

The primary infant outcomes assessed were malnutrition, diarrhea, and ARI. Malnutrition was assessed using the measurement of Middle-Upper Arm-Circumference (MUAC) and defined as infant with MUAC of ≤110 mm [41, 42]. The Integrated Management of Newborn and Childhood Illnesses (IMNCI) guideline was used to identify infants for diarrhea and ARI [43]. Diarrhea was defined as three or more episodes of loose stools in 24 h [44] and ARI was defined as a cough/cold accompanying fever or rapid breathing [45].

### Confounders

Potential confounding variables were identified using the modified disjunctive cause criterion. According to this criteria, covariates were considered to be potential confounders if: (1) they had significant associations with the exposure, the outcome or both; (2) they were not an instrumental variable; and (3) they were not likely to be on the causal pathway between the primary exposure and the outcome [46, 47]. Accordingly, the following confounders for both exposure and outcome were identified: family food access, maternal age, infant age, maternal education and occupational status, pregnancy intention, maternal service uptake, parity, maternal nutritional status, fear of giving birth, a history of chronic mental disorder, level of partner support, quality of partner relationship, social support, and stress coping ability. The “targeting step” in TMLE involves the use of these confounders to estimate the predicted probability of exposure (maternal depression) for each participant given these confounders. This probability is then used to update the estimated risk of the outcome, which is modelled using the observed exposure (depression/no depression) and the same set of confounders. The updated estimates of the risk of the outcome are then used to generate updated pairs of potential outcomes. The “average treatment effect (ATE)”, which here is the risk difference, is finally calculated as the average difference between these pairs across individual [28].

Social support during pregnancy was measured using the Oslo Social Support Scale (OSSS-3) [48]. The three items from OSSS-3 Likert scales were summed to a possible 14 points and women were categorized as having either ‘poor’ (total score < 9) or moderate to strong (overall rating 9–14) support. The Cronbach’s alpha for internal consistency was 0.76 in this study. The support that participants received from their partner was assessed using a 5-point Likert scale via the question ‘my partner helps me a lot’, which had possible responses; ‘always,’ ‘most of the time,’ ‘some of the time,’ ‘rarely,’ and ‘never’. Quality of partner relationship was assessed using a 3-point Likert scale via the question “How do you rate your relationship with your partner in day to day life?” with response categories, ‘very good’, ‘good’, and ‘poor’.

The maternal Middle-Upper Arm Circumference (MUAC) measured maternal nutritional status. The MUAC is validated for measuring nutritional status in the postnatal period and a cutoff score of 18-22 mm rated as ‘underweight’ and 22.5 to 31 mm as ‘normal” [49]. Participants were asked about their pregnancy intention via the question ‘at the time you became pregnant with this pregnancy, did you want to become pregnant, did you want to wait until later, or did you not want to have any more children?’. Their responses were categorized as ‘wanted now’, ‘wanted later’, and ‘not wanted at all’. The wanted now or later options were combined and labelled as ‘planned’ and ‘not wanted at all’ was labelled as ‘unplanned’. LBW was classified as a birth weight less than 2500 g [50].

The Perinatal Coping Inventory (PCI-4) was developed to assess maternal stress coping ability during pregnancy [51]. Coping styles within this tool included: (1) preparation for motherhood, ‘planned how you would handle the birth’; (2) avoidance ‘avoided being with people in general’; (3) positive appraisal ‘felt that being pregnant has enriched your life’; and (4) prayer ‘prayed that the birth would go well’. Participants were asked to report how often they used each of the above coping styles and responses were recorded using a 4-point Likert scale as 0 (never), (1) rarely, (2) sometimes, (3) most of the time [52]. The Cronbach’s alpha for internal consistency was 0.50 in this study.

### Statistical analysis

Completed survey data were downloaded from the Google cloud platform in an Excel spreadsheet, checked for completeness, and imported to Stata version 14 [53] for analysis. Descriptive statistics including mean (SD), median (IQR), frequency (percentage) were used as appropriate. Targeted maximum likelihood estimation (TMLE) was used to investigate the causal effects of perinatal depression on the risk of infant diarrhea, ARI and malnutrition using the estimated average treatment effect (ATE) [54, 55] which was reported as a risk difference (RD). The ATE estimates the average difference in the outcome between participants had they all been exposed and had they all been unexposed, adjusting for potential confounders [56]. TMLE applies G-computation and propensity score methods that involve both exposure and outcome mechanisms [28, 57], and is a doubly robust estimator, providing unbiased estimates when either the exposure model or the outcome model are miss-specified [58]. TMLE is also unbiased in the presence of outliers, unmeasured confounding, sparsity, and other modeling challenges [59]. Assumptions of the model includes no loss to follow up, similar to that for a randomized trial design [60]. We also assessed the potential mediating effects of LBW, postnatal depression, and early initiation of breast feeding using Generalized Structural Equation Models (GSEM) [61]. We included these same potential mediator variables in the causal model as a sensitivity analysis. Interaction terms for antenatal and postnatal depression with social support, partner support, and stress coping ability were also assessed for inclusion in the causal model.

A generalized estimating equation (GEE) model with a Poisson link function and exchangeable correlation structure was used as a comparison model to the TMLE model. Robust standard errors were used for the Poisson GEE given the clustering for the incidence of diarrhea, ARI and malnutrition within districts [62, 63]. Multicollinearity was assessed using correlation coefficients and the Variance Inflation Factor (VIF) with cut-off values of ≥0.8 and ≥ 10, respectively [64].

## Results

A total of 878 mother-infant dyads were followed to 6 months after birth. During follow up, two women withdrew and 10 were not contacted due to change of address leaving an overall loss-to-follow up of 1.3% and a final data set of 866 mother-infant dyads considered for analysis. We did a complete case analysis excluding the women who were lost from the cohort.

### Socio-demographic characteristics

The socio-demographic characteristics of the study participants are described in Table 1. The mean (SD) age of the mothers and the infants were 26.5 (4.5) years and 4.8 (1.3) months, respectively. There was a significant age difference for infants with and without diarrhea (*p* = 0.024) and malnutrition (*p* = < 0.001). Nearly half of the participants (*n* = 422, 48.7%) reported low-income, 322 (37.2%) completed high school, and 700 (80.8%) were Orthodox Christians. Education, income, and religion were associated with infant malnutrition and ARI (*p* < 0.001 for each). Most participants were engaged in home duties (*n* = 617, 71.3%), partnered (*n* = 832, 96.1%), and did not have difficulties in accessing food in the previous 3 months (*n* = 832, 96.1%).

**Table 1:** Socio-demographic characteristics of study participants according to those with and without Diarrhea, Acute Respiratory Infection (ARI), and Malnutrition in Gondar Town, Ethiopia, (*n* = 866)

Variable/category	Diarrhea (*n* = 147)		*p*-value	ARI (*n* = 187)		*p*-value	Malnutrition (*n* = 125)		*p*-value
	Yes, n (%)	No, n (%)		Yes, n, (%)	No, n (%)		Yes, n (%)	No, n (%)	
Women age at enrolment in years			0.571			0.057			0.947
18–24	45 (30.6)	236(32.8)		51 (27.3)	230(33.9)		39 (31.2)	242(32.7)	
25–34	89 (60.5)	436(60.6)		117 (62.6)	408(60.1)		77 (61.6)	448(60.5)	
> =35	13 (8.8)	47(6.5)		19 (10.1)	41(6.0)		9 (7.2)	51(6.8)	
Infant age (Mean(±SD) in months	4.98 (1.04)	4.73(1.29)	0.024	4.82 (1.21)	4.76(1.27)	0.543	4.09 (1.41)	4.9(1.19)	< 0.001
Household monthly income			0.942			0.793			0.022
Low	70 (47.6)	352(49.0)		87 (46.5)	335(49.3)		71 (56.8)	351(47.4)	
Medium	61 (41.5)	294(40.9)		80 (42.8)	275(40.5)		49 (39.2)	306(41.3)	
High	16 (10.9)	73(10.1)		20 (10.7)	69(10.2)		5 (4.0)	84(11.3)	
Women education			0.201			0.091			0.028
None	25 (17.0)	86(12.0)		33 (17.6)	78(11.5)		19 (15.2)	92(12.4)	
Primary	32 (21.8)	191(26.6)		43 (23.0)	180(26.5)		42 (33.6)	181(24.4)	
High school	50 (34.0)	272(37.8)		62 (33.2)	260(38.3)		45 (36.0)	277(37.4)	
Tertiary	40 (27.2)	170(23.6)		49 (26.2)	161(23.7)		19 (15.2)	191(25.8)	
Women occupation			0.079			0.661			0.101
Domestic duties	107 (72.8)	510(70.9)		133 (71.1)	484(71.3)		98 (78.4)	519(70.0)	
Student	4 (2.7)	9(1.2)		1 (0.5)	12(1.8)		2 (1.6)	11(1.5)	
Government employee	25 (17.0)	99(13.8)		28 (15.0)	96(14.1)		9 (7.2)	115(15.5)	
Self-employee	11 (7.5)	101(14.1)		25 (13.4)	87(12.8)		16 (12.8)	96(13.0)	
Women religion			0.155			0.000			0.467
Orthodox	125 (85.0)	575(80.0)		176 (94.1)	524(77.2)		104 (83.2)	596(80.4)	
Muslim	22 (15.0)	144(20.0)		11 (5.9)	155(22.8)		21 (16.8)	145(19.6)	
Women marital status			0.196			0.568			0.124
Single	3 (2.0)	31(4.3)		6 (3.2)	28(4.1)		8 (6.4)	26(3.5)	
Partnered	144 (98.0)	688(95.7)		181 (96.8)	651(95.9)		117 (93.6)	715(96.5)	
Difficulty accessing food in the last three months			0.299			0.258			0.124
Yes	8 (5.4)	26(3.6)		10 (5.3)	24(3.5)		8 (6.4)	26(3.5)	
No	139 (94.6)	693(96.4)		177 (94.7)	655(96.5)		117 (93.6)	715(96.5)	

*Note: p-value was based on chi-square test statistics*

### Maternal and infant characteristics

Maternal and infant characteristics of the study participants are described in Table 2. Most of the pregnancies were planned, 738 (85.2%), and 333 (38.4%) were first pregnancies. Almost all (95.7%) of the study participants had engaged with antenatal care (ANC), and most had attended postnatal care service (76.7%). Twenty-six (3.0%) and 128 (14.9%) were low birth weights and preterm births, respectively. Seventy-one (8.2%) infants were underweight, 529 (61.4%) mothers initiated early breastfeeding, and 577 (66.6%) strongly agreed that their infants were satisfied with breastfeeding. The incidence (95% CI) of diarrhea, ARI, and malnutrition were 17.0% (14.5–19.6), 21.6% (18.89–24.49), and 14.4% (12.2–16.9) respectively. During the follow up period, the episodes of ARI and diarrhea ranged from 1 to 5 and 1 to 4 respectively.

**Table 2:** Characteristics of maternal and infant participants to those with and without Diarrhea, Acute Respiratory Infection (ARI), and Malnutrition in Gondar Town, Ethiopia, (*n* = 866)

Variable/category	Diarrhea (Yes, *n* = 147)		*p*-value	(ARI) (Yes, *n* = 187)		*p*-value	Malnutrition (Yes, *n* = 125)		*p*-value
	Yes, n (%)	No, n (%)		Yes, n (%)	No, n (%)		Yes, n (%)	No, n (%)	
Pregnancy intention			0.562			0.397			0.688
Planned	123 (83.7)	615(85.5)		163 (87.2)	575(84.7)		108 (86.4)	630(85.0)	
Unplanned	24 (16.3)	104(14.5)		24 (12.8)	104(15.3)		17 (13.6)	111(15.0)	
Parity of the mother			0.793			0.675			0.432
1	53 (36.0)	280(38.9)		77 (41.2)			43 (34.4)	290(39.1)	
2	47 (32.0)	224(31.2)		55 (29.4)			45 (36.0)	226(30.5)	
3–8	47 (32.0)	215(29.9)		55 (29.4)			37 (29.6)	225(30.4)	
Antenatal care service uptake (at least one)			0.142			0.014			0.871
Yes	144 (98.0)	685(95.3)		185 (98.9)	644(94.8)		120 (96.0)	709(95.7)	
No	3 (2.0)	34(4.7)		2 (1.1)	35(5.2)		5 (4.0)	32(4.3)	
Postnatal care service			0.562			0.016			0.000
Yes	110 (74.8)	554(77.0)		131 (70.1)	533(78.5)		76 (60.8)	588(79.3)	
No	37 (25.2)	165(23.0)		56 (29.9)	146(21.5)		49 (39.2)	153(20.6)	
Low birth weight			0.016			0.000			0.452
Yes	9 (6.1)	20(2.4)		14 (7.0)	15(1.9)		5 (4.1)	22(2.8)	
No	138 (93.9)	699(97.6)		173 (93.0)	664(98.1)		120 (95.9)	719(97.2)	
Preterm birth			0.770			0.311			0.432
Yes	23 (15.7)	108(14.7)		33 (17.2)	97(14.2)		21 (17.2)	107(14.5)	
No	124 (84.3)	611(85.3)		154 (82.8)	582(85.8)		104 (82.8)	634(85.5)	
Exposure to coffee in pregnancy			0.429			0.065			0.075
Daily	64 (43.5)	293(40.7)		89 (47.6)	268(39.5)		58 (46.4)	299(40.4)	
Sometimes	44 (30.0)	255(35.5)		52 (27.8)	247(36.4)		32 (25.6)	267(36.0)	
Never	39 (26.5)	171(23.8)		46 (24.6)	164(24.1)		35 (28.0)	175(23.6)	
Exposure to cigarette in pregnancy			0.906			0.331			0.528
Yes	12(8.2)	78(10.8)		19(10.2)	71(10.5)		11(8.8)	79(10.7)	
No	135(91.8)	641(89.2)		168(89.8)	608(89.5)		114(91.2)	662(89.3)	
Nutritional status of the mother			0.193			0.109			0.186
Normal	131 (89.1)	664(92.3)		177 (94.6)	618(91.0)		111 (88.8)	684(92.3)	
Underweight	16 (10.9)	55(7.6)		10 (5.4)	61(9.0)		14 (11.2)	57(7.7)	
Early initiation of breast feeding			0.214			0.000			0.000
Yes	97 (66.0)	435(60.5)		152 (81.7)	380(55.8)		112 (89.3)	422(56.8)	
No	50 (34.0)	284(39.5)		35 (18.3)	299(44.2)		13 (10.7)	319(43.2)	
Care given for infant by			0.004			0.986			0.023
Mothers	136 (92.5)	701(97.5)		182 (97.3)	655(96.5)		125 (100.0)	712(96.1)	
Housekeeper	11 (7.5)	18(2.5)		5 (2.7)	24(3.5)		0 (0.0)	29(3.9)	
Maternal perception of infant response to breast feeding			0.110			0.279			0.000
Strongly agree	87 (59.2)	490(68.1)		132 (70.6)	445(65.5)		74 (59.2)	503(67.9)	
Agree	56 (38.1)	214(29.8)		53 (28.3)	217(32.0)		42 (33.6)	228(30.8)	
Not satisfied	4 (2.7)	15(2.1)		2 (1.1)	17(2.5)		9 (7.2)	10(1.3)	

*Note: p-value was based on chi-square test statistics*

Univariate associations were observed between diarrhea and LBW (*p* = 0.016) and infant care (*p* = 0.004). Univariate associations were also observed between ARI and ANC (*p* = 0.014), postnatal care (*p* = 0.016), LBW (*p* < 0.001), and early initiation of breastfeeding (*p* < 001). Early initiation of breast feeding (*p* < 0.001), postnatal care (*p* < 0.001), breastfeeding satisfaction (*p* < 0.001), and infant care (*p* = 0.024) were univariately associated with the risk of malnutrition.

### Psycho-social characteristics

The psycho-social characteristics of the study participants are described in Table 3. Fifty-six (6.5%) and 74 (8.5%) of the mothers had antenatal and postnatal depression respectively. In univariate analysis, neither antenatal or postnatal depression were associated with diarrhea, ARI or malnutrition (*p* > 0.1 for each). More than half of the study participants, 564 (66.6%), had a good relationship with their partners and 691 (79.8%) had good social support. About 403 (46.5%) women had frequent support from their partner and 547 (63.2%) had poor stress coping abilities. Diarrhea (*p* = 0.042) and ARI (*p* < 0.001) were associated with stress coping ability.

**Table 3:** Psycho-social characteristics of participants to those with and without Diarrhea, Acute Respiratory Infection (ARI), and Malnutrition in Gondar Town, Ethiopia, (*n* = 866)

Variable/category	Diarrhea (Yes, *n* = 147)		*P* value	ARI (Yes, *n* = 187)		*P* value	Malnutrition (Yes, *n* = 125)		*P* value
	Yes, (n, (%)	No, (n, (%)		Yes, (n, (%)	No, (n, (%)		Yes, (n, (%)	No, (n, (%)	
Marital relationship			0.776			0.052			0.946
Very good	35 (26.4)	167(24.3)		48 (25.5)	158(24.4)		29 (25.6)	176(24.5)	
Good	93 (66.0)	466(66.6)		122 (70.1)	432(65.5)		77 (66.1)	477(66.6)	
Poor	10 (7.6)	61(9.1)		8 (4.4)	64(10.1)		9 (8.3)	64(8.9)	
Social support			0.05			0.436			0.205
Good	126 (85.7)	565(78.6)		153 (81.8)	538(79.2)		105 (84.0)	586(79.1)	
Poor	21 (14.3)	154(21.4)		34 (18.2)	141(20.8)		20 (16.0)	155(20.9)	
Partner support			0.251			0.266			0.078
Always	74 (52.4)	318(45.3)		93 (49.7)	302(45.6)		60 (50.4)	332(45.9)	
Most of the time	37 (27.2)	201(29.1)		51 (27.3)	190(29.2)		34 (29.6)	204(28.6)	
Some of the time	18 (13.6)	136(19.7)		37 (19.8)	120(18.4)		12 (11.2)	140(20.0)	
Rarely	8 (6.8)	40(5.8)		6(3.2)	41(6.8)		11(8.8)	39(5.5)	
Stress coping ability			0.042			0.000			0.554
Good	65 (44.2)	254(35.3)		91 (48.7)	228(33.6)		49 (39.2)	270(36.4)	
Poor	82 (55.8)	465(64.7)		96 (51.3)	451(66.4)		76 (60.8)	471(63.6)	
Antenatal depression			0.197			0.714			0.225
Yes	6(4.1)	50(6.9)		11(5.9)	45(6.6)		5(4.0)	51(6.9)	
No	141(95.9)	669(93.1)		176(94.1)	634(93.4)		120(96.0)	690(93.1)	
Postnatal depression			0.140			0.559			0.135
Yes	8(5.4)	66(9.2)		14(7.5)	60(8.8)		15(12.0)	59(8.0)	
No	139(94.6)	653(90.8)		173(92.5)	619(91.2)		110(88.0)	682(92.0)	

*Note: p-value was based on chi-square test statistics*

### Causal association between depression and infant diarrhea, ARI, and malnutrition

Table 4 shows the results from the TMLE analysis. In the fully adjusted models, there was no association between antenatal depression and the risk of diarrhea, ARI or malnutrition. Specifically, compared to those without antenatal depression, the risk difference for antenatal depression and diarrhea was 0.8% (95%CI: − 9.2, 10.9), − 1.3% (95%CI: − 21.0, 18.5) for ARI, and − 7.3% (95%CI: − 22.0, 21.8) for malnutrition. Similarly, postnatal depression was not associated with diarrhea (risk difference = − 2.4%; 95%CI: − 9.6, 4.9), ARI (risk difference = − 3.2%; 95%CI: − 12.4, 5.9) or malnutrition (risk difference = 0.9%; 95%CI: − 7.6, 9.5). In comparison to the TMLE models, the point estimates for the IRR’s for each of the GEE models were generally much further from the null association i.e. an incidence rate ratio equal to one, although none reached statistical significance (Table 4).

**Table 4:** Estimates from TMLE showing the association of antenatal and postnatal depression with risk of Diarrhea, ARI, and Malnutrition in Gondar Town (*N* = 866), Ethiopia, 2018

	GEE model			TMLE
	Unadjusted IRR (95%CI)	Adjusted IRR (95% CI)	Adjusted *p*-value	djusted Risk Difference (95% CI)
**Diarrhea**				
Antenatal depression				
No	1.00	1.00		
Yes	0.65(0.29,1.45)	0.57(0.24,1.31)	0.185	0.8% (−9.2, 10.9)
Postnatal depression				
No	1.00	1.00		
Yes	0.85(0.46,1.60)	0.92(0.45,1.78)	0.811	-2.4% (−9.6, 4.9)
Adjusted for history of CMD, low birth weight, husband support, social support, stress coping ability, pregnancy condition, food access of the family, and maternal health care services.				
**Acute Respiratory Infection**				
Antenatal depression				
No	1.00	1.00		
Yes	0.85 (0.45,1.60)	0.92 (0.47,1.78)	0.798	−1.3% (−21.0, 18.5)
Postnatal depression				
No	1.00	1.00		
Yes	1.10(0.66,1.81)	1.00(0.58,1.72)	0.994	−3.2% (−12.4, 5.9)
Adjusted for age of the mother, age of the infant, maternal service uptake, fear to give birth, marital situation, husband support, social support, pregnancy need, food access, and occupation.				
**Malnutrition**				
Antenatal depression				
No	1.00	1.00		
Yes	0.47(0.16,1.40)	0.61(0.19,1.97)	0.407	−7.3% (−22.0, 21.8)
Postnatal depression				
No	1.00	1.00		
Yes	1.48(0.83,2.64)	1.43(0.71,2.89)	0.314	0.9% (−7.6, 9.5)
Adjusted for maternal services, parity, income, food access, occupation, educational status, marital situation, marital status, partner support, social support, history of common mental disorder, pregnancy condition, and nutritional status of the mother.				

In the mediation analysis using GSEM, results showed that: (i) There was no indirect effect of antenatal depression via postnatal depression on the risk of either diarrhea (*p* = 0.213), ARI (*p* = 0.660) or malnutrition (*p* = 0.182); (ii) There was no indirect effect of antenatal depression on the risk of malnutrition via LBW (*p* = 0.551) or early initiation of breastfeeding (*p* = 0.705). There were also no significant interactions between antenatal and postnatal depression with social support, partner support, and stress coping ability on the risk of diarrhea, ARI, and malnutrition (*p* > 0.2 for each). In the sensitivity analysis of the TMLE models, including the potential mediators did not change any of the substantive conclusions.

## Discussion

This study evaluated the incidence of diarrhea, acute respiratory infection (ARI), and malnutrition among infants age to 6 months in Gondar, Ethiopia and whether a causal relationship exists between perinatal depression and each of these outcomes. The incidence of diarrhea, ARI, and malnutrition was 17.0, 21.6, and 14.4%, respectively. Neither antenatal or postnatal depression appeared to be causally associated with the risk of diarrhea, ARI, and malnutrition.

The estimated incidence of diarrhea and ARI was slightly lower than that estimated in predominantly rural areas in Ethiopia, where there was a diarrhea and ARI incidence of 26.0 and 25% respectively [24]. These differences may be due to the method of measurement, the age of the included infants and the study setting. Diarrhea and ARI incidence are higher in rural areas compared to urban areas and increase with infant age [65]. Similarly, the incidence of malnutrition in the current study was lower than the 21.5% reported in a population-based cohort study conducted in other rural areas [22]. This could be due to differences in the screening tool used for malnutrition. In the current study, a MUAC of less than 110 mm was used as a cut of value [41, 42], indicating a sufficiently severe stage of malnutrition to put infants at considerable risk of death. Despite a substantial reduction of diarrhea, ARI, and malnutrition in Ethiopia in the last 15 years [66], the estimates from the current and previous studies demonstrate that these morbidities continue to be a significant public health problem in Ethiopia.

The main finding in this study was that antenatal depression was not causally associated with the risk of diarrhea, ARI, or malnutrition. This finding differs from previous Ethiopian community based cross-sectional [23] and cohort [24] studies which have reported significant associations between maternal common mental disorders (CMDs) and the risk of diarrhea and upper respiratory infection [20] but not ARI [21]. Elsewhere, a UK population based cohort study of children under the age of 4 years observed a 27% higher risk of episodes of lower respiratory tract infection amongst mothers that had experienced perinatal depression [67]. Similarly, antenatal depression predicted a higher risk of respiratory tract infections in offspring aged up to 10 months in Finland [68]. However, a meta-analysis of two prospective cohort studies demonstrated no association between antenatal depression and the risk of diarrhea [69]. Wasting, underweight, and stunting were also not associated with antenatal depression in Ethiopia in separate cross-sectional and cohort studies [22, 23].

Consistent with the above findings, this study found no causal association between postnatal depression and the risk of malnutrition, ARI, and diarrhea. Similarly, there was no association between CMDs during the postnatal period and underweight or stunting in Peru, Vietnam, India, and Ethiopia [70] and in two other community-based studies in Ethiopia [22, 23]. However, other studies in African countries have shown significant associations [10, 71–73]. Regarding risk of ARI and diarrhea, similar findings to our own were reported in community based cross-sectional [23] and cohort [24] studies in Ethiopia. However, postnatal depression did increase the risk of illness in infants aged four to 12 weeks and children under-5 years in prospective cohort studies conducted in Ghana [74] and Bangladesh [75] respectively. Further, a systematic review and meta-analysis reported postnatal depression increased the risk factor for diarrhea [69] and malnutrition [76], however, these reviews had an issue of heterogeneity in depression measurement. Thus, while the current and other studies in Ethiopia discount postnatal depression as a risk factor for diarrhea and ARI, studies in other African countries have observed associations. In the current study, unmeasured confounders such a sanitation, immunization, and lack of safe water had the potential to bias the estimates in either direction [66, 77]. However, the use of a doubly robust method will have helped reduce the risk of bias in comparison to previous studies that used standard regression techniques.

Despite a lack of clear evidence, various mechanisms have been suggested to explain an association between common mental disorders during the perinatal period and the risk of infant morbidity. For instance, common mental disorders during pregnancy can affect hypothalamic-pituitary-adrenocortical function, thereby influencing the development of the immune system in the offspring and subsequently increasing susceptibility to infections [78–80]. CMD might also negatively or positively affect the health-seeking behavior and lifestyle of the parents [81, 82]. However, we observed no evidence for a mediating effect for either low birth weight, early initiation of breastfeeding, or postnatal depression on any of the three outcomes. The lack of mediating effects was unsurprising given the lack of any overall association.

### Strength and limitations

This is the first prospective cohort study, conducted in an urban setting of Ethiopia, investigating the causal effect of antenatal and postnatal depression (measured using the EPDS) on the risk of diarrhea, ARI and malnutrition among infants aged under 6 months. We used Targeted Maximum Likelihood Estimation (TMLE) to assess the causal effects. Whilst there were several potential unobserved confounders such as personal hygiene, water access, latrine availability and cleanliness, various forms of violence, and immunization status, the method we used is doubly robust, such that model misspecification for either the exposure or outcome due to unobserved confounding reduces the potential for bias [58, 59].

The results of this study need to be considered under the assumptions for valid causal inference. This includes the positivity assumption, which states that all individuals have a positive probability of exposure. Since women with a high likelihood of having severe depression were excluded, this might have introduced a potential selection bias. In addition, the use of MUAC to assess malnutrition has not been validated in Ethiopia in infants of age under 4 months, and this might have either under or overestimated the true infant nutritional status. Similarly, we employed proxy indicators or symptoms (as established in the IMNCI guideline) to identify infants with diarrhea and ARI, which may have led to either under or over-diagnosis of cases. Nonetheless, the IMNCI guideline is recommended by the World Health Organization for use in primary health facilities and demonstrates high sensitivity and specificity.

## Conclusion

Our study provides evidence for a lack of any causal association between perinatal depression and the risk of adverse infant health outcomes within Ethiopia, including diarrhea, ARI and malnutrition. Previous associative studies may have overestimated the risk of poor health outcomes in infants amongst women with perinatal depression although further studies using similar causal inference methodology are required to confirm our findings.

## Supplementary Information

**Additional file 1.**

## Data Availability

The datasets used and/or analysed during the current study available from the corresponding author on reasonable request as this is part of a PhD work.

## Abbreviations

WHO: World health Organization
DSM-IV: Diagnostic and statistical manual of mental disorder
AND: Antenatal depression
EPDS: Edinburgh postnatal depression scale
SBRC: Social and behavioral research ethics committee
ODK: Open data collection kit
OSSS: Oslo social support scale
MUAC: Middle-upper arm circumference
PCI: Perinatal coping inventory
TLT: Tucker lewis index
CFI: Comparative fit index
RMSEA: Root mean square error of approximation
PHQ: Patient health questionnaire
BDI: Beck depression inventory
