# Supplementary material for: Effect of perinatal depression on risk of adverse infant health outcomes in mother-infant dyads in Gondar town: a causal analysis
Source: BMC Pregnancy Childbirth. 2021 Mar 26;21:255. doi: 10.1186/s12884-021-03733-5 (PMC7995776; doi:10.1186/s12884-021-03733-5)
Supplement: Supplementary file 1 — Additional file 1. [file 12884_2021_3733_MOESM1_ESM.docx]

**Estimating Effect of Perinatal Depression on risk of adverse infant health outcomes in mother-infant dyads in Gondar Town using the Targeted Maximum Likelihood Estimation Model**

Abel Fekadu Dadi (MPH) ^1, 2*^, Emma R Miller (PhD) ^2^, Richard J Woodman (PhD) ^3^, Telake Azale (PhD) ^4^, Lillian Mwanri (PhD)

# Annex 4: Questionnaire for collecting quantitative data

Flinders University, school of public health, English version questioner to assess perinatal depression and its adverse birth and infant health outcomes in Gondar Town, Northwest Ethiopia

## Annex 4a: Questionnaire for collecting quantitative data (1^st^ visit during pregnancy)

**Time required: 35’-40’**

Kebele name: ---------------------------------------------------------

Code of the mother: --------------------------------------------------

Name of the interviewer: ------------------------------------------------

Date of the interview: -------------------------------------------------------

Date of appointment for next visit -----------------------------------------

**Direction**: Please circle the response options and clearly fill open ended questions

| **Code** | **Variable** | **Response** | **Score/ explain** |
| --- | --- | --- | --- |
| **Part I**: **Socio-demographic characteristics of the women, now I will ask you about your self** | | | |
| 1.01 | Age of the mother | ------- years |  |
| 1.02 | Educational status of the mother | 1. No formal education 2. Grade 1-8 3. Grade 9-12 4. Diploma and above |  |
| 1.03 | Occupation of the mother | 1. Housewife 2. Student 3. Government employee 4. Self-employee |  |
| 1.04 | Marital status of the mother | 1. Single 2. Married 3. Divorced 4. Widowed 5. Separated |  |
| 1.05 | How you can explain your marital condition in general? | 1. Very good 2. Good 3. Bad 4. Very Bad |  |
| 1.06 | How often you discuss and agree with your husband on day to day life? | 1. Most of the time 2. Sometimes 3. Rarely 4. Never |  |
| 1.07 | Are you an active follower of any religion? | 1. Orthodox 2. Muslim 3. Catholic 4. Protestant 5. Other |  |
| 1.08 | In the last three months, have you ever worried that your household would not have enough food? | 1. Yes 2. No |  |
| 1.09 | Your monthly income in Ethiopian birr | -------------- |  |
| **Part II: Fertility: Now, I would like to ask you about the births that you have had in your life.** | | | |
| 2.01 | How many children do you have? | ------------ children |  |
| 2.02 | Is your current pregnancy a planned pregnancy? | 1. Yes, I wanted a child at this time 2. No, but I did want a child - later 3. No, I did not intend to have a child at all |  |
| 2.03 | How many months pregnant are you now? | -------------- weeks |  |
| 2.04 | Did you have difficulty in getting pregnant this time (e.g. used any fertility medication/waited long time to get pregnant? | 1. Yes 2. No |  |
| 2.05 | Mid upper arm circumference(MUAC) of the mother | -----------mm |  |
| **Part III. Feelings of depression (EPDS)**. **Tell us the way you have been feeling in the past (1) week including today. In the past seven days,** | | | |
| 3.01 | In the last week, have you been able to laugh and see the funny side of things? | As much as I always used to  Not as much as I used to  Certainly not as much as I used to  Not at all | 0  1  2  3 |
| 3.02 | In the last week, have you looked forward with enjoyment to things? | As much as I always used to  Rather less  Certainly less  Never looked forward | 0  1  2  3 |
| 3.03 | In the last week, have you blamed yourself unnecessarily when things went wrong? | Most of the time  Sometimes  Rarely  Never | 3  2  1  0 |
| 3.04 | In the last week, have you been anxious or worried for no good reason? | Most of the time  Sometimes  Not often  Never | 0  1  2  3 |
| 3.05 | In the last week, have you felt scared or panicky for no good reason? | Most of the time  Sometimes  Rarely  Never | 3  2  1  0 |
| 3.06 | In the last week, have things been getting on top of you? | Most of the time unable to cope  Sometimes unable  Mostly able  Coping as usual | 3  2  1  0 |
| 3.07 | In the last week, have you been so unhappy that you have had difficulty sleeping? | Most of the time  Sometimes  Rarely  Never | 3  2  1  0 |
| 3.08 | In the last week, have you felt sad or miserable? | Most of the time  Sometimes  Occasionally  Never | 3  2  1  0 |
| 3.09 | In the last week, have you felt so unhappy that you have been crying? | Most of the time  Sometimes  Occasionally  Never | 3  2  1  0 |
| 3.10 | In the last week, has the thought of harming yourself occurred to you? | Frequently  Sometimes  Not often  Never | 3  2  1  0 |
| 3.11 | Have you felt these symptoms before being pregnant? | 1. Yes 2. No |  |
| **Part IV. Oslo Social Support Scale (OSSS-3) to assess pregnant women social support,** Now, we would like to ask you questions about the support you get from different people | | | |
| 4.01 | How many people are so close to you that you can count on them if you have serious personal problems? | None  1 or 2  3 to 5  6 or more | 1  2  3  4 |
| 4.02 | How much concern do people show in what you are doing? | A lot of concern and interest  Some concern and interest  Uncertain  Little concern and interest  No concern and interest | 5  4  3  2  1 |
| 4.03 | How easy is it to get practical help from neighbours if you should need it? | Very easy  Easy  Possible  Difficult  Very difficult | 5  4  3  2  1 |
| 4.04 | My husband helps me a lot | Always  Most of the time  Some of the time  Rarely  Never | 5  4  3  2  1 |
| **Part V. Obstetric related factors, now I would like to ask you questions related to your current pregnancy** | | | |
| 5.01 | After you knew that you are pregnant, did you go anywhere to receive antenatal care? | 1. Yes 2. No |  |
| 5.02 | Including this pregnancy, for how many times have you been pregnant and give birth | 1. For the first time 2. Two and above | If “1” got Q. No 5.05 |
| 5.03 | Have you ever given birth to low weight baby? | 1. Yes 2. No |  |
| 5.04 | Have you ever given birth to preterm? | 1. Yes 2. No |  |
| 5.05 | Have you ever had a baby by cesarean delivery | 1. Yes 2. No |  |
| 5.06 | Do you have any fear of giving to this birth? | 1.Yes  2.No |  |
| 5.07 | Are you and your husband interested in the sex of your current pregnancy? | 1.Yes  2.No |  |
| 5.08 | Have you practice physical activity such as brisk walking, dancing, gardening, and usual housework for at least three hours/week | 1. Yes 2. No |  |
| 5.09 | How do you rate your daily health condition? | 1. Very good 2. Good 3. Bad 4. Very bad |  |
| 5.10 | Is there anybody who smoke near to you in your home or in your work place? | 1. Yes (exposure to second hand smoking) 2. No |  |
| 5.11 | How often are you drinking coffee during this pregnancy? | 1. Daily 2. Sometimes 3. Never |  |
| **Part VI. Stress coping ability of the women by Perinatal Coping Inventory(PCI-4)** | | | |
| 6.01 | Planned how you will handle the birth | Frequently  Sometimes  Not often  Never | 3  2  1  0 |
| 6.02 | Felt that being pregnant has enriched your life | Frequently  Sometimes  Not often  Never | 3  2  1  0 |
| 6.03 | Prayed that the birth will go well | Frequently  Sometimes  Not often  Never | 3  2  1  0 |
| 6.04 | Avoided being with people in general | Frequently  Sometimes  Not often  Never | 0  1  2  3 |

# Questionnaire for collecting Birth outcome and postnatal depression (2^nd^ visit, after delivery)

**Time required: 15- 20”**

**Direction**: Please circle the response options and clearly fill open ended questions

| Code | Questions | Response category | comment |
| --- | --- | --- | --- |
| **Part I. Adverse Pregnancy Outcome assessment questions** | | | |
| 1.01 | Birth weight of the new born | ----------- grams |  |
| 1.02 | Gestational weeks of the delivery | -----------weeks. days |  |
| 1.03 | Still birth event | 1. Yes 2. No |  |
| 1.04 | Mode of delivery | 1. Cs 2. Vaginal delivery |  |
| 1.05 | Type of delivery | 1. Single 2. Twin |  |
| 1.06 | Labor complication | 1. Yes 2. No |  |
| 1.07 | How many weeks since you have given birth? | -------------weeks |  |
| 1.08 | After you give birth, did you go anywhere to receive postnatal care? | 1. Yes 2. No |  |
| 1.09 | In the past 15 days how many days you were unable to perform your usual home activities? | --------------days |  |
| 1.10 | Weight of the mother | -------------------- kg |  |
| 1.11 | MUAC of the mother | -------------------cm |  |
| **Part II. Feelings of depression (EPDS)**. Tell us the way you have been feeling in the past (1) week including today. In the past seven days, | | | |
| 2.01 | In the last week, have you been able to laugh and see the funny side of things? For example: can you laugh at things which normally make you laugh? | As much as I always used to  Not as much as I used to  Certainly not as much as I used to  Not at all | 0  1  2  3 |
| 2.02 | In the last week, have you looked forward with enjoyment to things?  Another example is, are you able to look forward to market day? Or to something like this? | As much as I always used to  Rather less  Certainly less  Never looked forward | 0  1  2  3 |
| 2.03 | In the last week, have you blamed yourself unnecessarily when things went wrong? For example if your child gets ill do you blame yourself? Or, for example, if the crops fail? Or something like this? | Most of the time  Sometimes  Rarely  Never | 3  2  1  0 |
| 2.04 | In the last week, have you been anxious or worried for no good reason? | Most of the time  Sometimes  Not often  Never | 0  1  2  3 |
| 2.05 | In the last week, have you felt scared or panicky for no good reason? | Most of the time  Sometimes  Rarely  Never | 3  2  1  0 |
| 2.06 | In the last week, have things been getting on top of you? | Most of the time unable to cope  Sometimes unable  Mostly able  Coping as usual | 3  2  1  0 |
| 2.07 | In the last week, have you been so unhappy that you have had difficulty sleeping? | Most of the time  Sometimes  Rarely  Never | 3  2  1  0 |
| 2.08 | In the last week, have you felt sad or miserable? | Most of the time  Sometimes  Occasionally  Never | 3  2  1  0 |
| 2.09 | In the last week, have you felt so unhappy that you have been crying? | Most of the time  Sometimes  Occasionally  Never | 3  2  1  0 |
| 2.10 | In the last week, has the thought of harming yourself occurred to you? | Frequently  Sometimes  Not often  Never | 3  2  1  0 |
| **Part III. Oslo Social Support Scale (OSSS-3) to assess women social support after birth,**  Now, we would like to ask you questions about the support you get from different people | | | |
| 3.01 | How many people are so close to you that you can count on them if you have serious personal problems? | None  1 or 2  3 to 5  6 or more | 1  2  3  4 |
| 3.02 | How much concern do people show in what you are doing? | A lot of concern and interest  Some concern and interest  Uncertain  Little concern and interest  No concern and interest | 5  4  3  2  1 |
| 3.03 | How easy is it to get practical help from neighbours if you should need it? | Very easy  Easy  Possible  Difficult  Very difficult | 5  4  3  2  1 |
| 3.04 | My husband helps me a lot | Always  Most of the time  Some of the time  Rarely  Never | 5  4  3  2  1 |

##

## Annex 4c: Questionnaire for collecting data on infant health outcome (3^rd^ visit)

**Time required: 10’-15’**

| **Code** | **Questions** | **Response category** | **comment** |
| --- | --- | --- | --- |
| **Part I. Environmental factors** | | | |
| 1.01 | What is the age of the infant? | --------- months |  |
| 1.02 | Who is caring the infant most of the time? | 1. Mother 2. Father 3. Grand mother 4. Sister/brother 5. Home maid |  |
| 1.03 | My breasts seem to have enough milk | 1. Strongly agree 2. Agree 3. No idea 4. Disagree 5. Strongly disagree |  |
| 1.04 | My baby generally appears satisfied after breast feedings | 1. Strongly agree 2. Agree 3. No idea 4. Disagree 5. Strongly disagree |  |
| **Part II. Adverse Infant health outcome assessment questions** | | | |
| 2.01 | Breast feeding | 1. Exclusively breast fed 2. Non-exclusive breast fed |  |
| 2.02 | Malnutrition assessment | MUAC ----------- mm  Weight of the infant ------grams |  |
| 2.03 | Infant illness assessment | Diarrhea symptoms(three or more loose of stools in 24 hours)   1. Yes 2. No |  |
|  |  | ARI symptoms (cough/cold accompanying fever/or fast breathing)   1. Yes 2. No |  |
| 2.04 | How do you rate your infant daily health condition? | 1. Very good 2. Good 3. Neutral 4. Bad 5. Very bad |  |

## Annex 5b: Interview guide for health system administrators

**Time of the interview: 45’-50’**

Date of the interview ---------------------------

Code of the interviewee -------------------------

**Part I: Demographic information**

- 1. Age ----------years
  2. Total experience as health worker and health system administrator ---------------- years
  3. Profession 1. Clinical nurse 2. Midwifery 3. Health officer 4. Medical doctor
  4. Marital status 1. Never married 2. Married (living together) 3. Separated 4. Widowed
  5. Monthly income : ---------------ETB
  6. Religion 1. Orthodox 2. Catholic 3. Muslim 4. Protestant

**Part II: Interview guide**

1. **Knowledge and practice about perinatal depression**

- How would you describe a mother of good mental health? Probe
- They feel good, can do their usual work, they can come for service, can understand what they told
- What do you think perinatal depression is? Probe, Mental illness, mood disorder,
- Who is at risk of depression? Probe,

Everybody, pregnant women, postnatal women, adolescents

- What are the sign and symptoms the women with depression could show? Probe
- Dissatisfaction, think of worthlessness, hate to do their usual activity, think of suicide, feeling sad, feeling tired
- What do you think about the cause of depression? Probe
- Poverty, pregnancy, lack of support, fear of birth
- When would the mother develop depression? Probe
- Immediately after pregnancy, 1^st^ trimester, 2^nd^ trimester, 3^rd^ trimester, after birth
- What do you think should be done for the mother with severe depression? probe
- Nothing, refer to hospital, counseling
- What would happen if depressed women cannot gate appropriate intervention? Probe
- Effect on birth outcome, service uptake, maternal health consequence, may die, may suicide

1. **Health system administrators opinion about health care system concerning perinatal depression**

- How our country policy looks about perinatal depression as a problem?
- Do our health system have plan, strategy, and initiative to screen depression during perinatal period?
- What activities have been undertaken to facilitate screening for perinatal depression? Mainstreaming the problem, training health professionals, including in a plan, evaluating the performance of planned activities
- Would you tell me how could health professionals identify women with depression? Probe
- Sign and symptom, from their complain,
- When do you think it is favorable to screen women with depression? Probe
- During ANC, PNC, during vaccination time, during delivery
- Where do you think it is favorable to screen women with depression? Probe
- At health facility, at their home, during campaign
- What could health professionals use to identify women who have depression sign? Probe

Observation, screening tool, laboratory,

- If health professionals want to screen and treat depressed women who come to their department, can they do? What do they need to do so?
- Is the condition/set up of the health facility initiate you/them to do so? Probe
- Facility to treat women with depression, trained professionals, time to screen
- What do you think is a barrier for screening and treating women with depression/ for health professionals? Probe
  - No way in place for screening, no guideline for treatment, no system emplaced to do this, I don’t know how to identify and treat

**I thank you very much again for your participation in the interview and providing responses, and will strictly be kept confidential.**
